# Media for Coping During COVID-19 Social Distancing: Stress, Anxiety, and Psychological Well-Being

**DOI:** 10.3389/fpsyg.2020.577639

**Published:** 2020-12-18

**Authors:** Allison L. Eden, Benjamin K. Johnson, Leonard Reinecke, Sara M. Grady

**Affiliations:** ^1^Department of Communication, Michigan State University, East Lansing, MI, United States; ^2^Department of Advertising, University of Florida, Gainesville, FL, United States; ^3^Department of Communication, Johannes Gutenberg University Mainz, Mainz, Germany

**Keywords:** stress, anxiety, media, coping, well-being, COVID-19, students

## Abstract

In spring 2020, COVID-19 and the ensuing social distancing and stay-at-home orders instigated abrupt changes to employment and educational infrastructure, leading to uncertainty, concern, and stress among United States college students. The media consumption patterns of this and other social groups across the globe were affected, with early evidence suggesting viewers were seeking both pandemic-themed media and reassuring, familiar content. A general increase in media consumption, and increased consumption of specific types of content, may have been due to media use for coping strategies. This paper examines the relationship between the stress and anxiety of university students and their strategic use of media for coping during initial social distancing periods in March-April 2020 using data from a cross-sectional survey. We examine links between specific types of media use with psychological well-being concepts, and examine the moderating roles of traits (hope, optimism, and resilience) as buffers against negative relationships between stress and anxiety and psychological well-being. Our findings indicate that stress was linked to more hedonic and less eudaimonic media use, as well as more avoidant and escapist media-based coping. Anxiety, on the other hand, was linked to more media use in general, specifically more eudaimonic media use and a full range of media-based coping strategies. In turn, escapist media was linked to negative affect, while reframing media and eudaimonic media were linked to positive affect. Avoidant coping was tied to poorer mental health, and humor coping was tied to better mental health. Hedonic and need-satisfying media use were linked to more flourishing. Hope, optimism, and resilience were all predictive of media use, with the latter two traits moderating responses to stress and anxiety. The findings give a nuanced portrait of college students’ media use during a pandemic-induced shutdown, showing that media use is closely intertwined with well-being in both adaptive and maladaptive patterns.

## Media for Coping During COVID-19 Social Distancing: Stress, Anxiety, and Psychological Well-Being

In the spring of 2020, COVID-19 concerns drove American universities to cancel face-to-face classes, which resulted in millions of residential college students leaving campus mid-semester with no plan to return (Hess, 2020). This decision led to uncertainty, concern, and stress for students, as they were urged to remain sequestered in their primary residences. The University of Washington suspended face-to-face instruction on March 7, and Harvard followed March 10. Students at Michigan State University were informed on March 11 that all face-to-face instruction would be suspended. By March 14, classes were confirmed to be online for the remainder of the semester, and students were strongly encouraged to return to their permanent residences^1^. Across the United States, universities and states were making similar decisions: University of Florida also suspended face-to-face classes on March 11, and by March 17 had sent all students who were able to return home back to their primary residences^2^. March Madness, a popular inter-collegiate basketball tournament, was canceled, and commencements across the country postponed. By the end of March, over 14 million college students’ education had been suddenly altered by protective measures to counter COVID-19 (Hess, 2020).

During this same period, video streaming increased sharply, especially during daytime hours (Weissbrot, 2020). Early indications suggest that the pandemic altered media use patterns. Popular press articles suggested that viewers were either seeking out pandemic-themed media (Sutton, 2020) or turning to reassuring, familiar content (MRC Data, 2020). This increase in media consumption, or the consumption of specific types of content, may have been due to the use of media as a *coping strategy* to deal with stress and anxiety experienced during the initial social distancing period. In this paper we examine the relationship between the stress and anxiety of university students and their strategic use of media for coping during initial social distancing periods. We further associate specific media coping factors with psychological well-being outcomes, and examine the moderating factors of trait hope, optimism, and resilience as buffers against negative outcomes from psychological stress during the pandemic.

### Stress and Coping

Psychological stress is many-faceted, but usually stems from a disconnect (or disequilibrium) between one’s available resources and the demands they face (Lazarus, 1966; Folkman et al., 1986). Stress can result from many contextual factors, from impending threats and future worries, to existing harm and ongoing challenges; stress can then lead to many negative psychological and physiological outcomes such as unhealthy behaviors and increased anxiety (Segrin, 1999; Hudd et al., 2000). How individuals attempt to manage stress is known as coping (Carver and Connor-Smith, 2010). Coping is multi-dimensional and encompasses both problem-focused and emotion-focused strategies (Lazarus and Folkman, 1984). Problem-focused coping focuses on the stressor itself, whereas emotion-focused coping focuses on affective responses to the stressor, often through avoidance, escapism, or distraction. These disengagements are frequently considered ineffectual, while problem-focused coping, positive reappraisal, and meaning creation reliably predict positive emotional outcomes (Folkman and Moskowitz, 2000).

Along with many others, one population suddenly facing unexpected stress due to COVID-19 countermeasures were the suddenly relocated (at least, moved online) United States university students. In March 2020, many American residential universities moved classes online, sent students away from residential facilities, and shut down or minimized capacity of residence halls to protect students, employees, and staff against COVID-19 (Hess, 2020). The stresses of quarantine and social isolation are known to have negative psychological effects, including heightened stress and anxiety (Brooks et al., 2020; Pfefferbaum and North, 2020; Tsamakis et al., 2020). In addition to disease-related concerns for themselves and their loved ones, the disruption of daily life and routine during stressful events may lead to functional impairment and post-traumatic stress outcomes (Pat-Horenczyk et al., 2006). Preliminary evidence also indicates that college students reporting increased anxiety during initial COVID-19 outbreaks were concerned not only about the infection itself, but about the economic and academic impact of COVID on their futures (Cao et al., 2020). When confronted with stress, individuals seek support from social networks, hobbies, and leisure activities (Kitzrow, 2003). But during this initial March period of COVID-19 precautions, many states enacted “Stay Home Stay Safe” orders, closing all businesses, recreation, and entertainment not deemed essential to supporting life (e.g., State of Michigan, Executive Office of the Governer [Gretchen Whitmer], 2020). This had a two-fold effect of removing entertainment and hobby outlets for stress, and further isolating students. Given the activities that can be safely indulged in at home, media use seems to be a common and prolific avenue for stress reduction, as well as one that can be safely engaged in while social distancing. A survey of young adults in the UK with mental health needs found that media were a critical source of coping for those especially negatively impacted by the lack of social contact and support (Young Minds, 2020). Thus, media use may be an important avenue of coping with stress and anxiety, particularly one that can be engaged while remaining sequestered at home.

### Media and Coping

Wolfers and Schneider (2020) recently identified three major lines of research investigating media and coping: (a) media as a stress coping tool, (b) media as mood management, and (c) problematic media use as a form of dysfunctional attempts at coping. The coping literature has primarily focused on media as a dysfunctional coping mechanism (e.g., Carver and Connor-Smith, 2010; Müller et al., 2016). However, other research suggests that, depending on the type of content and the surrounding environment, media can be an effective coping mechanism. For example, Nabi et al. (2017) found that media use is a primary coping strategy for people facing health or academic stress, and individuals under high stress are likely to turn to media for relaxation and recovery (Anderson et al., 1996; Reinecke and Eden, 2016). Media use broadly has been demonstrated to reduce stress (Nabi et al., 2017; Prestin and Nabi, 2020), help alleviate anxiety (e.g., Khoo and Oliver, 2013; Perks, 2019), and ultimately foster positive psychological well-being outcomes (Reinecke and Eden, 2016).

In terms of problem-focused coping, specifically, adolescents who reported stress in specific domains (e.g., parents, peers, appearance) preferred to watch talk shows on these topics (Trepte et al., 2001). Similarly, Knobloch-Westerwick et al. (2009) found individuals elect to spend more time with information that is relevant to successfully navigating areas of life where they were experiencing stress. Both these responses suggest people use media to approach or define a problem as a form of coping. In terms of emotion-focused coping, a large body of literature has addressed media use as a form of escapism (e.g., Katz and Foulkes, 1962; Halfmann and Reinecke, 2021). Such research suggests that media exposure is frequently used to seek distraction from frustration, stress, and anxiety in everyday life (e.g., Kubey and Csikszentmihalyi, 1990; Moskalenko and Heine, 2003). While escapist media use, similar to avoidance-oriented and emotion-focused coping in general, is frequently discussed as a dysfunctional coping strategy (e.g., Meier et al., 2018), other conceptualizations suggests that escapism through media use can be a functional short-term strategy, in that it may temporarily help the individual reduce stress and anxiety, and prepare for subsequent problem-focused coping attempts (Halfmann and Reinecke, 2021).

Emotion-focused forms of coping via media may be particularly relevant in the context of the COVID-19 crisis. The coping literature suggests that emotion-focused coping strategies are particularly effective and functional if the individual has low control over the situation and stressor, making problem-focused coping difficult or even impossible (Lazarus and Folkman, 1987; Eatough and Chang, 2018). As the spread of COVID-19 represents a global pandemic, emotion-focused coping attempts via media use may be particularly likely. Previous research also suggests that media exposure is a particularly common coping tool when other coping resources are limited or unavailable. For example, Mares and Cantor (1992) found that lonely individuals turned to portrayals of other lonely individuals for coping. Similarly, Hofer and Eden (2020) found that individuals experiencing decreased social support and opportunity for relationship building were more dependent on media to compensate for missed social connections than those who enjoyed strong social support. Additionally, research on stress recovery demonstrates that entertaining media content is particularly used for stress relief when social support is unavailable (Reinecke, 2009a, b). Taken together, this body of literature suggests that during the COVID-19 pandemic—when confronted with limited control on external problem-solving measures to combat their new-found stressors—students may be more likely to employ emotion-focused coping tactics via media use.

During social distancing, students were isolated from their friends and routine, as well as concerned about changes in the local pandemic status, and therefore we might expect that stress and anxiety would be heightened during social distancing, and that crucial coping resources, such as the availability of social support, will be largely absent or impaired. As such, if users are turning to media to cope with negative feelings, we may see overall increases in media use. At the same time, media can be used as part of various and even competing coping strategies: for some users, media may play a role in problem-focused coping, where they turn to media to keep monitoring the local situation or to learn about other pandemics. On the other hand, users may feel a need to distance themselves from the current situation, and focus instead on the emotional benefits of media. The first aim of the present study was to examine the relationships between stress and anxiety resulting from social distancing and the use of media exposure within a variety of well-established coping strategies (Carver, 1997). Beyond that, the literature on media use and psychological well-being has identified a number of specific psychological mechanisms that may connect media use to positive psychological outcomes (for an overview, see Reinecke and Oliver, 2016). In the following sections, we will review a selection of these mechanisms which are then integrated in our hypothesized model.

### Media and Mood

One central mechanism that connects media use to psychological well-being is the mood-altering effects of media exposure. A large number of studies in the tradition of mood management theory (Zillmann and Bryant, 1985; Zillmann, 1988) demonstrate that entertaining media in particular can be used to positively influence or manage negative moods (Knobloch and Zillmann, 2002; Knobloch, 2003). This may occur even when the mood is brought about by cyclical hormonal shifts (Meadowcroft and Zillmann, 1987). Therefore, individuals experiencing significant negative mood changes may be more likely to consume media to attempt to change their mood.

Beyond mood valence, entertainment research distinguishes content based on hedonic versus eudaimonic motivations for media consumption (cf. Oliver and Raney, 2011). Hedonic motivations are primarily pleasure-seeking, and lead to positive affective experiences typically associated with traditional and formulaic genres of media entertainment, such as comedy, action movies, or crime series. In line with Nabi and Krcmar (2004), we posit that the positive emotions associated with such hedonic forms of media enjoyment can have short-term psychological benefits, and this may be associated with stress-related coping. In this case, we might expect that hedonically motivated media usage will increase during social distancing.

Eudaimonic motivations, on the other hand, are concerned with existential questions of purpose in life, meaning, or moral values. These motives often lead to more contemplative and emotionally complex media selections and experiences, and are often associated with exposure to somber or poignant media content. Previous research suggests that such forms of eudaimonic entertainment may provide important role models for dealing with critical life events (Greenwood and Long, 2015), as they often portray protagonists that show perseverance and positive adaptation to adversity, thus providing opportunities for the vicarious experience of meaning making and successful coping (Slater et al., 2018). As a consequence, the desire to gain insight and seek meaning in these uncertain times may also lead to increased eudaimonic media use during social distancing.

### Media and Intrinsic Need Satisfaction

Another avenue of media research demonstrates that entertainment media can satisfy intrinsic needs. Intrinsic needs are universal human drives which benefit individuals, such as being competent, having autonomy over one’s own life, and feeling a deep sense of connection in personal relationships (self-determination theory; Ryan and Deci, 2000; Vansteenkiste et al., 2020). Prior literature has demonstrated that entertainment can satisfy these needs in a number of ways (Tamborini et al., 2010; Reinecke et al., 2012). Moreover, basic psychological need satisfaction has been linked to the use of both interactive media, such as video games or social media (e.g., Ryan et al., 2006; Reinecke et al., 2014; Johnson et al., 2020) and non-interactive media, such as movies, TV series, or video clips (e.g., Adachi et al., 2018; Granow et al., 2018). In the context of social distancing, media users are stuck at home often in relative isolation, with external limits on their ability to travel or work, and with little personal agency in combating a global pandemic. Therefore, the public may have limited access to other avenues in which to feel competent, autonomous, and socially connected. Media perceived to satisfy these needs may therefore motivate media use and may be particularly appealing to users. For example, users may report increased social networking usage to remain in contact with their friends and support network (cf. Sheldon et al., 2011).

In sum, then, we predict that (H1) stress and (H2) anxiety will have positive associations with (a) quantity of media exposure, (b) using media to cope, (c) hedonic media use, (d) eudaimonic media use, and (e) intrinsically satisfying media use.

### Effects on Affect, Mental Health, and Flourishing

While stress and coping may shift patterns of media consumption and gratifications, we also sought to explore how media use may be influencing users’ self-reports of psychological well-being more generally. All forms of media use discussed above (and addressed in H1 and H2) have been linked to psychological well-being in previous research (Reinecke and Oliver, 2016). Extant work on media use and coping clearly suggests that media exposure is a frequently used tool for stress coping and can significantly facilitate the coping process (Wolfers and Schneider, 2020). Furthermore, both exposure to hedonically and eudaimonically motivated media use has been linked to well-being benefits. Hedonic media entertainment has primarily associated with increased well-being in the form of increased positive and decreased negative affect (for an overview, see Reinecke, 2017), while eudaimonic entertainment has also been identified as a source of more complex forms of psychological well-being, such as feeling self-transcendent emotions such as elevation, awe, or gratitude (e.g., Oliver et al., 2018; Janicke-Bowles et al., 2019). Finally, the satisfaction of the basic psychological needs for autonomy, competence, and relatedness, both in general and via media use specifically, has been linked to various psychological well-being indicators (e.g., Johnson et al., 2020; Vansteenkiste et al., 2020). In sum, these findings suggest that all forms of media use addressed in the present study have the potential to show beneficial effects on different facets of media users’ well-being.

The present study examines the association of media use with three different indicators of psychological well-being: the presence of positive affect and absence of negative affect as an indicator of subjective well-being (Diener et al., 1999); the absence of psychological symptoms as an indicator of mental health (Ware and Sherbourne, 1992); and flourishing as an indicator of psychological functioning in different areas of life (Diener et al., 2010). Therefore, we predict that (H3) quantity of media exposure, (H4) using media to cope, (H5) hedonic media use, (H6) eudaimonic media use, and (H7) intrinsically satisfying media use will have positive associations with (a) affect, (b) mental health, and (c) flourishing.

We clearly are not suggesting that media use fully mediates the relationship connecting stress and anxiety with well-being. On the contrary, stress and anxiety are important factors in psychological well-being more generally. However, we do suggest that media use (and particularly coping-based, emotionally motivated, and need-satisfying media consumption) will influence this relationship, such that media use which serves to support coping and need satisfaction will reduce the effect of stressors on well-being, as follows:

Stress and anxiety will have negative total and direct effects on affect, mental health, and flourishing, but (H8) positive mediation effects via (a) quantity of media exposure, (b) using media to cope, (c) hedonic media use, (d) eudaimonic media use, and (e) intrinsically satisfying media use will partially suppress the negative influences of stress and anxiety on (i) affect, (ii) mental health, and (iii) flourishing.

### Moderating Traits

Numerous protective factors, however, may alter both the initial stress reaction as well as the ways in which entertaining media are used as coping tools. In the psychological literature, such factors are frequently discussed in the context of resilience. The theoretical concept of resilience refers to positive adaptation after adversity (Fletcher and Sarkar, 2013; Kalisch et al., 2017). Adversity can occur both in the form of chronic, long-lasting, and systemic stressors (such as ongoing abuse), or in the form of acute stressors, (including isolated events such as personal loss or changes in life conditions; Pangallo et al., 2015). Furthermore, adversity may refer both to severe and traumatic life events but also to more common and less disruptive stressors such as daily hassles (Fletcher and Sarkar, 2013; Chmitorz et al., 2020).

Two theoretical perspectives differentiate resilience as either a relatively stable trait or a dynamic process (Fletcher and Sarkar, 2013; Pangallo et al., 2015). The trait perspective treats resilience as a tendency to show positive adaptation to adversity in various situations and contexts of life (e.g., Smith et al., 2008), whereas the state perspective suggests that resilience describes the dynamic process of a successful response to a specific stressor or life event (Aburn et al., 2016). In addition to general trait resilience, several individual difference variables are known to facilitate positive adaptation to stressors and adversity, and these are often referred to as resiliency factors (Windle, 2011; Pangallo et al., 2015).

One key resiliency factor is the presence and cultivation of positive emotions and affect (Fletcher and Sarkar, 2013; Pangallo et al., 2015). In this context, two variables have frequently been identified as resiliency factors: optimism and hope (Gillespie et al., 2007; Pangallo et al., 2015). Both variables have consistently been linked to positive adaptation and increased psychological well-being (Alarcon et al., 2013). While both optimism and hope represent forms of positive affect, they are distinct theoretical concepts. Previous work has defined optimism as positive “generalized outcome expectancies” (Scheier and Carver, 1985, p. 219), suggesting that optimists have a global expectation that positive things will happen to them. Hope, in contrast, refers to an individual’s perceived ability to find ways to pursue their goals (i.e., pathways) and to show the necessary perseverance to follow those routes to reach their goals (i.e., agency) (Snyder, 2002). Put differently, people may feel optimistic for various reasons (e.g., because they believe in their own abilities or simply in their own luck), whereas hope more strongly emphasizes the subjective perception of possessing the competencies necessary for shaping a positive future (Alarcon et al., 2013).

In the context of the present study, both general trait resilience as well as more specific resilience factors such as optimism and hope appear relevant for multiple reasons. First, previous research suggests a direct positive influence of trait resilience and protective and promotive resiliency factors on adaptation to stress and psychological well-being (Windle, 2011). Therefore, trait resilience, hope, and optimism may show a negative main effect on perceived stress in response to social distancing and a positive main effect on psychological well-being indicators such as flourishing. Second, the resilience literature further suggests that these factors may also moderate the effects of a stressor on well-being, as they promote positive adaptation to adversity (Windle, 2011; Kalisch et al., 2017). This suggests that the stress and anxiety resulting from the COVID-19 situation may have a smaller detrimental effect on the psychological well-being and flourishing of those individuals with higher levels of trait resilience, optimism, and hope. Finally, the presence or absence of resiliency factors may also influence whether and how individuals use media during COVID-19 related social distancing.

While the empirical evidence on the interplay of media use and resilience factors is very limited, a number of theoretical mechanisms connect both concepts (Reinecke and Rieger, 2021). Initial evidence suggests that resiliency factors, such as trait optimism, may influence the individual preference for hedonic versus eudaimonic media content (Oliver and Raney, 2011). In turn, exposure to media content may also strengthen resiliency, by eliciting feelings of hope for example (Prestin and Nabi, 2020). Furthermore, previous research suggests trait resilience significantly influences individual coping styles, revealing a positive correlation between trait resilience and active coping and positive reframing and a negative correlation with behavioral disengagement, denial, and self-blame (Smith et al., 2008). Whether these patterns also apply to media use for coping, however, has not yet been demonstrated. Additionally, resiliency factors may also moderate the relationships of stress and anxiety with media use, and of media use with psychological well-being, respectively. Research on media use for stress coping demonstrates that the presence or absence of other coping resources, such as social support, moderates the effects of daily hassles on the frequency of media use for stress coping (Reinecke, 2009a). The resiliency factors in the present study may show similar interaction effects on the relationships between stress and anxiety, media use, and psychological well-being.

**FIGURE 1:**
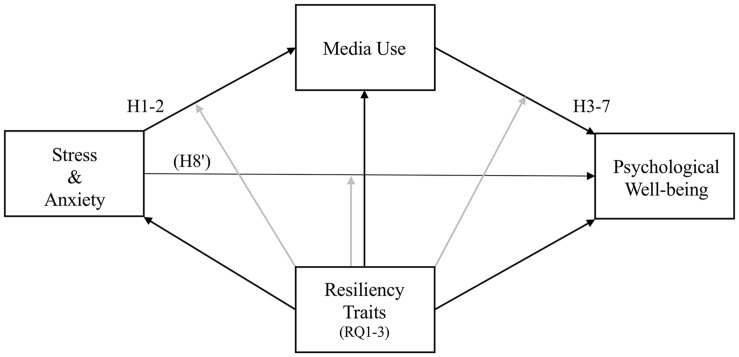
Conceptual model. Stress and anxiety are tested as two distinct independent variables. H3-7 predict different forms of media use (media exposure, using media to cope, hedonic media use, eudaimonic media use, and intrinsically satisfying media use) will be associated with three well-being measures (affect, mental health, and flourishing). H8 predicts media use will mediate the relationship between stress/anxiety and well-being. RQ1-3 examine the main effects and potential moderation role of resiliency trait factors hope, optimism, and resilience.

To explore the role of trait resiliency factors in the interplay of stress, media use, and well-being, we pose the following research questions: Do (RQ1) trait optimism, (RQ2) trait hope, and (RQ3) trait resilience have main effects on stress, anxiety, media use, and affect, mental health, and flourishing, and do they moderate hypothesized effects? Our conceptual model appears in Figure 1.

## Materials and Methods

To test the hypotheses and research questions, students at two American universities that canceled face-to-face instruction due to COVID-19 were surveyed. Both universities canceled face-to-face instruction the week of March 9, 2020, and students completed a cross-sectional survey between March 23, 2020 and April 17, 2020. The study preregistration, data, and materials are available at https://osf.io/ktwrn/. All procedures and measures were approved by the ethical board of each university.

### Participants

An initial 459 students accessed the questionnaire. Screening criteria removed 29 incomplete cases as well as 5 cases that reported more than 24 h per day on a single media activity. This left *N* = 425 for analysis. Participants were aged *M* = 20.19, *SD* = 2.18; 68.5% were women; 11.5% self-identified as Hispanic or Latino, 78.1% White/Caucasian, 12.7% Asian, 8.5% Black/African-American, 1.2% Native American, 0.5% Pacific Islander, 2.1% Other (multiple selections possible); 25.9% freshmen, 27.8% sophomores, 25.9% juniors, 18.6% seniors, and 1.9% senior +.

### Measures

All non-trait items were framed with instructions referring to “your feelings and thoughts since social distancing began.” Descriptives for all measures are reported in Table 1^3^.

**TABLE 1:** Descriptive statistics for study variables.

Variable	M	SD	Min	Max	α	Skew	Kurtosis
Stress	3.099	0.480	1.43	4.86	.797	0.110	0.352
Anxiety	2.339	0.812	1.00	4.00	.905	0.266	−0.723
Media Exposure	21.416	11.562	0.00	120.00*	−	2.563	14.618
Problem-Focus Media Coping	2.087	0.792	1.00	4.00	.822	0.418	−0.648
Avoidant Media Coping	1.681	0.738	1.00	4.00	.823	1.069	0.342
Escapist Media Coping	2.649	0.772	1.00	4.00	.827	−0.143	−0.829
Reframing Media Coping	2.547	0.853	1.00	4.00	.765	−0.053	−0.731
Humor Media Coping	2.228	0.885	1.00	4.00	.670	0.281	−0.857
Hedonic Media	5.411	1.080	1.00	7.00	.910	−0.817	0.773
Eudaimonic Media	4.333	1.359	1.00	7.00	.881	−0.225	−0.439
Media Need Satisfaction	4.413	1.249	1.00	7.00	.930	−0.568	0.246
Optimism	3.322	0.709	1.17	5.00	.758	−0.025	−0.189
Hope	3.058	0.453	1.38	4.00	.842	−0.223	0.421
Resilience	3.226	0.754	1.00	5.00	.805	−0.186	−0.008
Affect	3.328	0.703	1.00	5.00	.897	−0.331	−0.149
Mental Health	3.155	0.724	1.00	5.00	.810	−0.234	−0.136
Flourishing	5.314	0.992	2.00	7.00	.913	−0.361	−0.292

*A total of n = 112 reported media exposure > 24 h in a typical day. Given multitasking possibilities, we only excluded those reporting more than 24 h for a single medium. Median value for media exposure is 19.00 h.

The two independent variables were stress and anxiety in the context of social distancing. Stress was measured with the 14-item Perceived Stress Scale (Cohen et al., 1983), e.g., “how often have you been upset because of something that happened unexpectedly?” *Never* (1) to *Very Often* (5). Anxiety was measured with the 7-item Generalized Anxiety Disorder scale (Spitzer et al., 2006), which assesses how often one was “bothered” by problems, e.g., “Feeling nervous, anxious, or on edge,” *Not at all* (1) to *Nearly every day* (4).

Media variables included media exposure^4^, various coping-focused uses of media, and subjective entertainment experiences. Media exposure was assessed by participants’ self-reports of hours spent in an “average day” since social distancing began on each of the following: television, movies, radio and music, internet websites, video social media, social media, video conferences, phone calls, video games, books, podcasts, and instant messaging. Participants were instructed to use decimal points for fractions of hours, and report 0 h if a media type was not typically used. The sum of all media exposure was computed.

Coping via media was measured with the 28-item Brief COPE (Carver, 1997), adapted to refer to “media use” as a component of each coping tactic [e.g., “I’ve been turning to media to take my mind off things,” *I haven’t been doing this at all* (1) to *I’ve been doing this a lot* (4)]. A planned exploratory factor analysis (EFA) with maximum likelihood extraction and direct oblimin rotation was used to identify dimensions of media coping. EFA found five factors with eigenvalues > 1. Items with loadings below 0.5 were omitted (with the exception of one item loading 0.497). The factors represented distinct coping dimensions with good face and content validity, of problem-focused, avoidant, escapist, reframing, and humor-based coping (Table 2). These are consistent with literature on coping and media, so we treat these five dimensions as distinct variables.

**TABLE 2:** Exploratory factor analysis for media coping.

Item	Original dimension	Media dimension	Factor loading
**23. I’ve been using media to try to get advice or help from other people about what to do.**	**Instrumental Support**	**Problem-Focus**	**.849**
**10. I’ve been getting help and advice from other people through media.**	**Instrumental Support**	**Problem-Focus**	**.681**
**25. I’ve been using media to think hard about what steps to take.**	**Planning**	**Problem-Focus**	**.549**
**14. I’ve used media to try to come up with a strategy about what to do.**	**Planning**	**Problem-Focus**	**.510**
5. I’ve been using media to get emotional support from others.	Emotional Support	Problem-Focus	.471
27. I’ve been using media as a kind of mediation or prayer.	Religion	Problem-Focus	.359
20. I use media to help accept the reality of the fact that this has happened.	Acceptance	Problem-Focus	.357
22. I try to find comfort, meaning, or spirituality through media.	Religion	Problem-Focus	.345
2. I’ve been using media to do something about the situation I’m in.	Active Coping	Problem-Focus	.315
**8. I use media because I refuse to believe what’s been happening.**	**Denial**	**Avoidant**	**.894**
**3. I’ve been using media to tell myself this isn’t real.**	**Denial**	**Avoidant**	**.828**
**16. I’ve used media because I’ve given up the attempt to cope.**	**Behavioral Disengagement**	**Avoidant**	**.538**
**6. I’ve used media because I give up trying to deal with things.**	**Behavioral Disengagement**	**Avoidant**	**.497**
26. I use media to blame myself for things that happened.	Self-Blame	Avoidant	.444
**1. I’ve been turning to media to take my mind off things.**	**Self-Distraction**	**Escapist**	**.734**
**4. I’ve been using media to make myself feel better.**	**Substance Use**	**Escapist**	**.673**
**9. I’ve been using media to let my unpleasant feelings escape.**	**Venting**	**Escapist**	**.577**
**11. I’ve been using media to help me get through it.**	**Substance Use**	**Escapist**	**.563**
19. I’ve been using media to think about the situation less.	Self-Distraction	Escapist	.488
7. I’ve been using media to try to make the situation better.	Active Coping	Escapist	.409
24. I’ve used media as I’m learning to live with the situation.	Acceptance	Escapist	.395
**17. I’ve used media to look for something good in what is happening.**	**Positive Reframing**	**Reframing**	**.797**
**12. I’ve been using media to try seeing things in a different light, to make the situation seem more positive.**	**Positive Reframing**	**Reframing**	**.596**
15. I’ve been getting comfort and understanding from media.	Emotional Support	Reframing	.295
**28. I’ve been making fun of the situation through media use.**	**Humor**	**Humor**	**.630**
**18. Media are useful for making jokes about the situation.**	**Humor**	**Humor**	**.571**
21. I express my negative feelings through media use.	Venting	Humor	.390
13. I use media to criticize myself.	Self-Blame	Humor	.361

*Items in bold included in final dimensions. Factor loadings from pattern matrix.*

Frequencies of consuming media perceived to meet hedonic motivations (6 items; e.g., “Lets me have fun”) and eudaimonic motivations (6 items; e.g., “Makes me more reflective”) were measured on a 7-point scale, *Not at all* (1) to *Very much* (7). Intrinsic need satisfaction via media was measured with a 12-item version (La Guardia et al., 2000; Reinecke et al., 2014) of the Basic Psychological Need Satisfaction scale (BPNS; Ilardi et al., 1993), probing media content that made one feel, e.g., “free to be who I am,” *Not at all* (1) to *Very much* (7).

With regard to moderating traits, three established scales were administered. Trait optimism was measured with the 6-item Life Orientation Scale Revised (Scheier et al., 1994), e.g., “In uncertain times, I usually expect the best,” *Strongly disagree* (1) to *Strongly agree* (5). Trait hope was measured with the 8-item Hope Scale (Snyder et al., 1991), e.g., “There are lots of ways around any problem,” *Definitely false* (1) to *Definitely true* (4). Trait resilience was measured with the 6-item Brief Resilience Scale (Smith et al., 2008), e.g., “I tend to bounce back quickly after hard times,” *Strongly disagree* (1) to *Strongly agree* (5).

Finally, affect, mental health, and flourishing outcomes were assessed with a set of established measures. Affect was measured with the 12-item Scale of Positive and Negative Experience (SPANE; Diener et al., 2010), which assesses frequency of experience different feelings (e.g., “Joyful,”), *Very seldom or never* (1) to *Very frequently or always* (5). Negative items (e.g., “Afraid”) were reverse coded to allow for combination with positive items in a general affect measure. Mental health was measured with the 5-item mental health subscale of the SF-36 (Ware and Sherbourne, 1992), which assesses frequency of experiences, e.g., “You felt calm and peaceful,” *Never* (1) to *Constantly* (4). Flourishing was measured with the 8-item Flourishing Scale (Diener et al., 2010), e.g., “I lead a purposeful and meaningful life,” *Strongly disagree* (1) to *Strongly agree* (7).

### Analysis Plan

Descriptive statistics and correlations are presented as a preliminary analysis. To test initial hypotheses, regression analyses tested the effects of the following variables in three blocks: (a) demographics, (b) trait moderators, and (c) state stress and anxiety, on five dependent variables of media use (media exposure, coping, hedonic, eudaimonic, and intrinsically satisfying). Given the multidimensional nature of media-based coping from our EFA, effects were examined for each of the five dimensions of media coping separately. A fourth block was used to enter interaction terms between trait moderators (one trait at a time) and stress and anxiety (labeled as block 4a/b/c in Tables 4, 5).

**TABLE 3:** Correlations among study variables.

Variable	(1)	(2)	(3)	(4)	(5)	(6)	(7)	(8)	(9)	(10)	(11)	(12)	(13)	(14)	(15)	(16)
(1) Stress																
(2) Anxiety	.619***															
(3) Media Exp.	.090	.185***														
(4) Prob. Cope	.145**	.254***	.073													
(5) Avoid. Cope	.328***	.402***	.132**	.464***												
(6) Escap. Cope	.398***	.536***	.200***	.440***	.474***											
(7) Refram. Cope	.087	.221***	.114*	.520***	.273***	.480***										
(8) Humor Cope	.144**	.240***	.121*	.379***	.370***	.444***	.395***									
(9) Hedonic	.114*	.193***	.043	.057	.006	.360***	.265***	.245***								
(10) Eudaimonic	−.140**	.033	.094	.388***	.142**	.166***	.303***	.176***	.235***							
(11) Need Satisfaction	−.021	.068	.107*	.393***	.191***	.307***	.319***	.271***	.312***	.507***						
(12) Optimism	−.389***	−.273***	−.045	−.075	−.211***	−.105*	.089	−.051	.107*	.101*	.060					
(13) Hope	−.220***	.039	.149**	.052	−.091	.099*	.195***	.027	.324***	.220***	.245***	.456***				
(14) Resilience	−.429***	−.331***	−.043	−.057	−.142**	−.226***	.045	−.102*	−.016	.151**	.007	.490***	.348***			
(15) Affect	−.639***	−.633***	−.111*	−.094	−.334***	−.389***	.021	−.128**	−.009	.201***	.121*	.362***	.235***	.378***		
(16) MentalHealth	−.722***	−.695***	−.139**	−.165***	−.427***	−.426***	−.065	−.104*	−.058	.118*	.027	.423***	.210***	.414***	.758***	
(17) Flourishing	−.326***	−.186***	.073	.017	−.121*	.003	.184***	.013	.318***	.308***	.344***	.443***	.548***	.322***	.394***	.355***

N = 425. *p < .05, **p < .01, ***p < .001.

**TABLE 4:** Associations of stress and anxiety with media use variables.

Predictors	Media exposure	Hedonic media	Eudaimonic media	Media need satisfaction
	
	β	β	β	β
Block 1: Demographics	Δ*R*^2^ = .043	Δ*R*^2^ = .003	Δ*R*^2^ = .015	Δ*R*^2^ = .011
Woman	.078	.036	−.069	−.041
Latinx	−.010	.003	−.029	−.026
White	−.192***	.018	−.087	−.043
Age	−.012	−.027	.049	−.066
Education	−.028	.048	−.049	−.016
Block 2: Traits	Δ*R*^2^ = .042	Δ*R*^2^ = .129	Δ*R*^2^ = .056	Δ*R*^2^ = .076
Optimism	−.109	.023	−.031	−.008
Hope	.229***	.378***	.215***	.300***
Resilience	−.059	−.150**	.081	−.100
Block 3: IVs	Δ*R*^2^ = .018	Δ*R*^2^ = .028	Δ*R*^2^ = .018	Δ*R*^2^ = .001
Stress	−.016	.129*	−.167*	−.012
Anxiety	.160*	.093	.159*	.046
Main Effects Model *R*^2^	.103	.160	.090	.088
Block 4a: Moderation	Δ*R*^2^ = .019	Δ*R*^2^ = .003	Δ*R*^2^ = .002	Δ*R*^2^ = .006
Optimism × Stress	.127*	.025	.016	−.079
Optimism × Anxiety	−.188**	−.074	.030	.103
Block 4b: Moderation	Δ*R*^2^ = .004	Δ*R*^2^ = .000	Δ*R*^2^ = .002	Δ*R*^2^ = .000
Hope × Stress	−.018	.002	−.008	−.008
Hope × Anxiety	.074	.002	.055	.012
Block 4c: Moderation	Δ*R*^2^ = .001	Δ*R*^2^ = .002	Δ*R*^2^ = .006	Δ*R*^2^ = .001
Resilience × Stress	.027	.043	.004	−.021
Resilience × Anxiety	−.051	−.056	.073	.051

Regression models with hierarchical entry. Standardized coefficients for each block are reported from the model in which that block was first added. N = 422. *p < .05, **p < .01, ***p < .001.

**TABLE 5:** Associations of stress and anxiety with media coping.

Predictors	Problem-focus coping	Avoidant coping	Escapist coping	Reframing coping	Humor coping
	
	β	β	β	β	β
Block 1: Demographics	Δ*R*^2^ = .013	Δ*R*^2^ = .012	Δ*R*^2^ = .039	Δ*R*^2^ = .024	Δ*R*^2^ = .002
Woman	−.080	.029	.173***	.135**	−.030
Latinx	.016	−.026	−.084	−.026	.005
White	−.058	−.104*	−.024	.020	−.017
Age	−.064	.000	−.055	.059	.004
Education	.027	−.015	.035	−.091	.032
Block 2: Traits	Δ*R*^2^ = .019	Δ*R*^2^ = .046	Δ*R*^2^ = .083	Δ*R*^2^ = .036	Δ*R*^2^ = .018
Optimism	−.085	−.196***	−.070	.009	−.026
Hope	.134*	.020	.231***	.192***	.092
Resilience	−.070	−.052	−.255***	−.014	−.128*
Block 3: IVs	Δ*R*^2^ = .064	Δ*R*^2^ = .152	Δ*R*^2^ = .199	Δ*R*^2^ = .042	Δ*R*^2^ = .049
Stress	.040	.128*	.133*	−.003	.023
Anxiety	.263***	.364***	.424***	.233***	.238***
Main Effects Model *R*^2^	.095	.211	.320	.102	.069
Block 4a: Moderation	Δ*R*^2^ = .023	Δ*R*^2^ = .012	Δ*R*^2^ = .004	Δ*R*^2^ = .014	Δ*R*^2^ = .015
Optimism × Stress	.152**	.119*	.051	.120*	.128*
Optimism × Anxiety	−.039	−.043	.010	−.033	−.036
Block 4b: Moderation	Δ*R*^2^ = .004	Δ*R*^2^ = .004	Δ*R*^2^ = .001	Δ*R*^2^ = .001	Δ*R*^2^ = .005
Hope × Stress	.067	−.004	.026	.026	.018
Hope × Anxiety	−.020	.067	−.013	−.004	.062
Block 4c: Moderation	Δ*R*^2^ = .015	Δ*R*^2^ = .023	Δ*R*^2^ = .009	Δ*R*^2^ = .010	Δ*R*^2^ = .020
Resilience × Stress	.136*	.157**	.051	.115*	.124*
Resilience × Anxiety	−.068	−.051	.045	−.067	−.001

Regression models with hierarchical entry. Standardized coefficients for each block are reported from the model in which that block was first added. N = 422. *p < .05, **p < .01, ***p < .001.

Next, regression analyses tested the effects of the following variables in four blocks: (a) demographics, (b) trait moderators, (c) state stress and anxiety, and (d) media use on the dependent variables of affect, mental health, and flourishing. An additional fifth block was used to enter interaction terms between trait moderators (one trait at a time) and, stress, anxiety, and media use (labeled as block 5a/b/c in Table 6).

**TABLE 6:** Associations of media use with affect, mental health, and flourishing.

Predictors	Affect	Mental health	Flourishing
	
	β	β	β
Block 1: Demographics	Δ*R*^2^ = .046	Δ*R*^2^ = .072	Δ*R*^2^ = .009
Woman	−.170***	−.235***	.014
Latinx	.090	.053	.037
White	−.029	−.003	−.029
Age	.107	.129*	.031
Education	−.105	−.138*	−.103
Block 2: Traits	Δ*R*^2^ = .168	Δ*R*^2^ = .208	Δ*R*^2^ = .356
Optimism	.216***	.307***	.230***
Hope	.050	−.017	.429***
Resilience	.237***	.238***	.068
Block 3: Stress/Anxiety	Δ*R*^2^ = .319	Δ*R*^2^ = .368	Δ*R*^2^ = .026
Stress	−.320***	−.385***	−.120*
Anxiety	−.422***	−.418***	−.092
Block 4: Media Use	Δ*R*^2^ = .046	Δ*R*^2^ = .029	Δ*R*^2^ = .085
Media Exposure	−.033	−.035	.021
Problem−Focus Coping	.005	.007	−.072
Avoidant Coping	−.057	−.157***	.020
Escapist Coping	−.162***	−.078	−.041
Reframing Coping	.136**	.046	.064
Humor Coping	.002	.106**	−.031
Hedonic Media	.037	.003	.157***
Eudaimonic Media	.094*	.048	.094
Need Satisfaction	.076	.007	.180***
Main Effects Model *R*^2^	.580	.678	.476
Block 5a: Moderation	Δ*R*^2^ = .017	Δ*R*^2^ = .008	Δ*R*^2^ = .011
Optimism × Reframing	−.100*	−.062	−.061
Block 5b: Moderation	Δ*R*^2^ = .018	Δ*R*^2^ = .012	Δ*R*^2^ = .033
Hope × Anxiety	−.085	−.050	−.145**
Hope × Humor	−.005	−.018	.124**
Block 5c: Moderation	Δ*R*^2^ = .005	Δ*R*^2^ = .005	Δ*R*^2^ = .008
Resilience	−	−	−

Regression models with hierarchical entry. Standardized coefficients for each block are reported from the model in which that block was first added. Only select interactions from Blocks 5a/b/c are presented here, in the interest of space. The ΔR^2^ for each moderation block includes interactions between moderator of interest and stress, anxiety, and all nine media use variables, for a total of 11 interaction terms. Multicollinearity was not a threat, as all interaction terms showed tolerance > .858. N = 422. *p < .05, **p < .01, ***p < .001.

**FIGURE 2:**
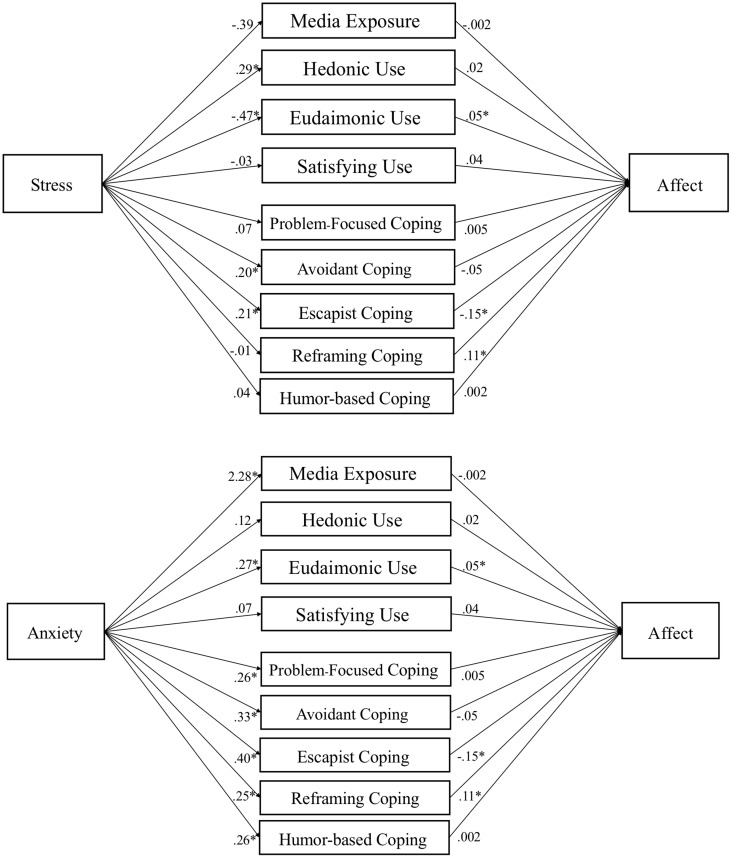
Media use partially mediates influence of stress and anxiety on affect. Note. Parallel mediation of media motives on affect. Gender, age, ethnicity, race, level of education, and traits (hope, optimism, and resilience) are used as covariates in PROCESS Model 4 using 10,000 bootstrap samples. Path coefficients reported are unstandardized, *p* < .05 denoted with an *. Indirect effects appear in text and Table 7.

**FIGURE 3:**
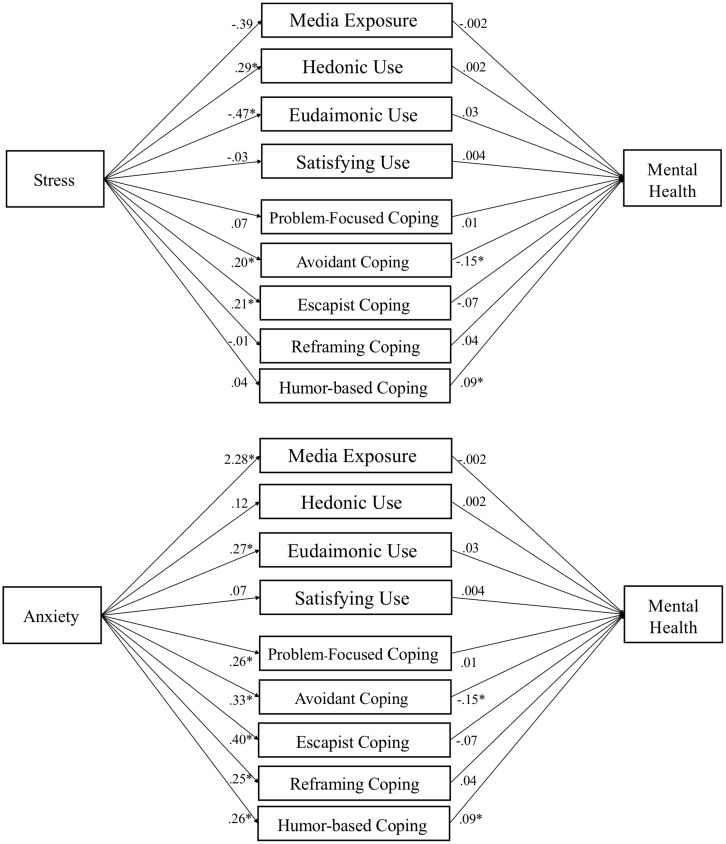
Media use partially mediates influence of stress and anxiety on mental health. *Note.* Parallel mediation of media motives on mental health. Gender, age, ethnicity, race, level of education, and traits (hope, optimism, and resilience) are used as covariates in PROCESS Model 4 using 10,000 bootstrap samples. Path coefficients reported are unstandardized, *p* < .05 denoted with an *. Indirect effects appear in text and Table 7.

**FIGURE 4:**
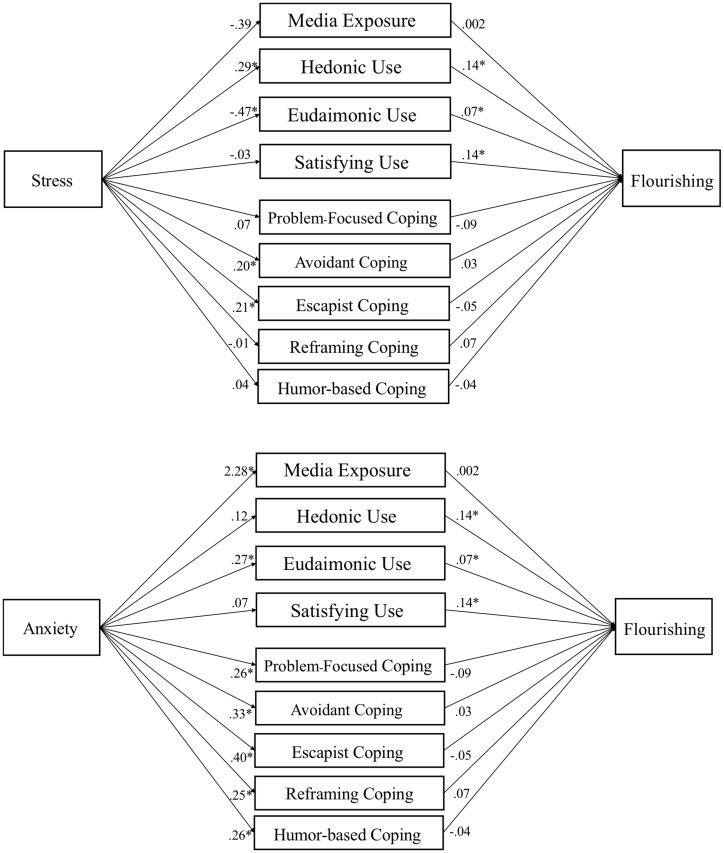
Media use partially mediates influence of stress and anxiety on flourishing. Note. Parallel mediation of media motives on flourishing. Gender, age, ethnicity, race, level of education, and traits (hope, optimism, and resilience) are used as covariates in PROCESS Model 4 using 10,000 bootstrap samples. Path coefficients reported are unstandardized, *p* < .05 denoted with an *. Indirect effects appear in text and Table 7.

The mediation hypotheses were tested with the PROCESS macro (Hayes, 2018). Demographics and traits were included as covariates. Media use variables were tested as nine parallel mediators (five coping dimensions, plus media exposure, hedonic, eudaimonic, and need satisfaction). Given two IVs and three DVs, six mediation models were tested (see Figures 2-4).

Finally, trait moderators from the earlier regression analyses were tested as moderators of the mediation effects using PROCESS.

## Results

### Preliminary Analyses

Descriptive statistics appear in Table 1 and correlations among study variables are presented in Table 3.

### Relationships With Media Use

Table 4 presents the relationship of stress and anxiety, controlling for demographics and traits, with media exposure, hedonic media use, eudaimonic media use, and media need satisfaction. These analyses test H1a/c/d/e and H2a/c/d/e (i.e., excluding media coping). We find that stress is associated with more hedonic media use and less eudaimonic media use. In contrast, anxiety is associated with more eudaimonic media use, as well as more media exposure.

In Table 5, the tests of H1b and H2b are presented, examining how stress and anxiety relate to the five identified dimensions of media coping. Stress was positively associated with avoidant and escapist coping via media. Anxiety had medium-sized positive associations with all five dimensions of media coping.

### Relationships With Affect, Mental Health, and Flourishing

Table 6 reports regression models for the effects of stress and anxiety, as well as media variables, on affect, mental health, and flourishing, in order to test H3 through H7. Both stress and anxiety had substantial negative correlations with affect and mental health. Stress also was negatively correlated with flourishing.

Media exposure was not linked to differences in affect, mental health, or flourishing. Examining the effects of media coping dimensions, escapist coping was associated with less positive affect, and reframing coping with more positive affect. Avoidant coping was associated with lower mental health scores, but humor coping with higher mental health scores. This suggests that different media-related coping strategies were associated with different indicators of well-being, potentially suggesting adaptive or maladaptive functions.

Eudaimonic media use was connected to more positive affect, and both hedonic media and media need-satisfaction were associated with higher levels of flourishing.

### Media Use Mediates Stress and Anxiety’s Effects on Well-Being

Mediation tests (H8) found a mix of positive indirect effects, which were hypothesized to suppress the negative effects of stress and anxiety on well-being outcomes. Specifically, reframing coping suppressed the effect of anxiety on affect, β = .032, *SE* = .013, 95% CI [.009, .061]. Humor coping suppressed the effect of anxiety on mental health, β = .025, *SE* = .012, 95% CI [.006, .051]. Eudaimonic media suppressed the effects of anxiety on affect, β = .015, *SE* = .009, 95% CI [.001, .036] and flourishing, β = .015, *SE* = .010, 95% CI [.000, .037].

We also found some negative indirect effects, suggesting that some (maladaptive) forms of media use may be associated with negative effects on well-being. Specifically, escapist coping mediated the effects of stress on affect, β = −.022, *SE* = .013, 95% CI [−.051, −.002] and anxiety on affect, β = −.069, *SE* = .024, 95% CI [−.120, −.027]. Avoidant coping mediated effects of stress on mental health, β = −.020, *SE* = .011, 95% CI [−.043, −.0003], and anxiety on mental health, β = −.057, *SE* = .016, 95% CI [−.092, −.028]. Eudaimonic media mediated the effect of stress on affect, β = −.016, *SE* = .010, 95% CI [−.039, −.005]. For path models of each test see Figures 2-4.

### Moderation by Trait Resilience, Optimism, and Hope

After accounting for demographics, traits demonstrated some influence on both media use and well-being, supporting our research questions, as shown in Block 2 of Tables 4-6. Optimism was negatively associated with avoidant coping, and positively with affect, mental health, and flourishing. Hope was positively associated with media exposure; problem-focused, escapist, and reframing forms of coping; hedonic, eudaimonic, and need-satisfying media use; and flourishing. Resilience was negatively associated with escapist and humor coping, hedonic media, and was positively associated with affect and mental health.

To examine the research questions’ interaction effects, the regression models reported in Tables 4-6 were extended beyond their main effect models to include moderating traits (one per extended model) as moderators of the effects of IVs.

Optimism positively moderated the effect of stress on media exposure, but negatively moderated the effect of anxiety on media exposure (Table 4). Pessimists observed stronger effects of anxiety on media exposure. Optimism also positively moderated the effect of stress on problem-focused coping, avoidant coping, reframing coping, and humor coping (Table 5). Pessimists under stress were less likely to use media for problem-focused coping, reframing coping, or humor coping, while optimists under stress were more likely to use media for problem-focused coping, avoidant coping, or humor coping. Additionally, an interaction between trait optimism and reframing coping was tied to less positive affect (Table 6). More pessimistic individuals had more negative effects of reframing on affect.

Hope negatively moderated the effect of anxiety on flourishing, and hope positively moderated the effect of humor coping on flourishing (Table 6). Hopeful individuals showed less flourishing in response to anxiety. Individuals scoring low in hope had negative effects of humor coping on their flourishing.

Resilience positively moderated the effect of stress on problem-focused, avoidant, reframing, and humor coping (Table 5). Resilient individuals under stress were more likely to use media for problem-focused coping, avoidant coping, and humor coping. Less resilient individuals under stress were less likely to use media for avoidant coping, reframing coping, or humor coping.

### Traits Moderate the Mediation

Finally, we considered how traits might moderate the observed mediation effects. The significant instances of moderated mediation are probed and presented in Table 8. First, we examined how traits might interact with stress and anxiety to influence media use and subsequent well-being. Specifically, optimism moderated the indirect effect of stress on affect via reframing coping, *index* = 0.028, *SE* = 0.016, 95% CI [0.003, 0.065]. Those very low on optimism showed mediation via less reframing, and those very high on optimism showed suppression via more reframing via media. Optimism moderated the indirect effect of stress on mental health via avoidant coping, *index* = −0.051, *SE* = 0.020, 95% CI [−0.094, −0.016]. Optimists (1 *SD* above the mean optimism score) showed a negative mediation effect: Their stress led to avoidant coping, which was then linked to lower mental health. In contrast, optimism moderated the indirect effect of stress on mental health via humor coping, *index* = 0.024, *SE* = 0.013, 95% CI [0.004, 0.056]: Extremely stressed pessimists had decreased humor media use, while extreme optimists had greater humor media use which suppressed the effect of stress on mental health.

**TABLE 7:** Summary of hypothesis testing.

Prediction	Description	Supported	Details
H1a	Stress → Media Exposure	No	
H1b	Stress → Media Coping	Partial	Yes for avoidant and escapist dimensions
H1c	Stress → Hedonic	Yes	
H1d	Stress → Eudaimonic	No	Effect in opposite direction
H1e	Stress → Need Satisfaction	No	
H2a	Anxiety → Media Exposure	Yes	
H2b	Anxiety → Media Coping	Yes	
H2c	Anxiety → Hedonic	No	
H2d	Anxiety → Eudaimonic	Yes	
H2e	Anxiety → Need Satisfaction	No	
H3a	Media Exposure → Affect	No	
H3b	Media Exposure → Mental Health	No	
H3c	Media Exposure → Flourishing	No	
H4a	Media Coping → Affect	Partial	Yes for reframing; opposite effect for escapist
H4b	Media Coping → Mental Health	Partial	Yes for humor; opposite effect for avoidant
H4c	Media Coping → Flourishing	No	
H5a	Hedonic → Affect	No	
H5b	Hedonic → Mental Health	No	
H5c	Hedonic → Flourishing	Yes	
H6a	Eudaimonic → Affect	Yes	
H6b	Eudaimonic → Mental Health	No	
H6c	Eudaimonic → Flourishing	No	
H7a	Need Satisfaction → Affect	No	
H7b	Need Satisfaction → Mental Health	No	
H7c	Need Satisfaction → Flourishing	Yes	
H8a-i	Stress/Anxiety → Media Exp. → Affect	No	
H8b-i	Stress/Anxiety → Media Coping → Affect	Partial	Yes for anxiety via reframing. Opposite effect for stress and anxiety via escapist coping.
H8c-i	Stress/Anxiety → Hedonic → Affect	No	
H8d-i	Stress/Anxiety → Eudaimonic → Affect	Partial	Yes for anxiety. Opposite effect for stress.
H8e-i	Stress/Anxiety → Need Satisf. → Affect	No	
H8a-ii	Stress/Anxiety → Media Exp. → Mental Health	No	
H8b-ii	Stress/Anxiety → Media Coping → Mental Health	Partial	Yes for anxiety via humor. Opposite effects for stress and anxiety via avoidant coping.
H8c-ii	Stress/Anxiety → Hedonic → Mental Health	No	
H8d-ii	Stress/Anxiety → Eudaimonic → Mental Health	No	
H8e-ii	Stress/Anxiety → Need Satisf. → Mental Health	No	
H8a-iii	Stress/Anxiety → Media Exp. → Flourishing	No	
H8b-iii	Stress/Anxiety → Media Coping → Flourishing	No	
H8c-iii	Stress/Anxiety → Hedonic → Flourishing	No	
H8d-iii	Stress/Anxiety → Eudaimonic → Flourishing	Partial	Yes for anxiety.
H8e-iii	Stress/Anxiety → Need Satisf. → Flourishing	No	

RQ1	Optimism → or X	Main effects: Optimism ↓ avoidant, ↑ affect, mental health, flourishing. Interaction effects: Optimism × stress ↑ media exposure, problem-focus, avoidant, reframing, humor. Optimism × anxiety ↓ media exposure. Optimism × reframing ↓ affect.	
RQ2	Hope → or X	Main effects: Hope ↑ media exposure, problem-focus, escapist, reframing, hedonic, eudaimonic, need satisfaction, flourishing. Interaction effects: Hope × anxiety ↓ flourishing. Hope × humor ↑ flourishing.	
RQ3	Resilience → or X	Main effects: Resilience ↓ hedonic, escapist, humor, ↑ affect, mental health. Interaction effects: Resilience × stress ↑ problem-focus, avoidant, reframing, humor.	

*All predictions in H1-H8 were for positive associations.*

**TABLE 8:** Significant moderated mediation models.

Moderator level	Mediation effect	Indirect effect	SE	95% CI
Optimism				
+1 SD	Stress→Reframing→Affect	0.016	0.018	[−0.018, 0.054]
Mean	Stress→Reframing→Affect	−0.008	0.016	[−0.045, 0.212]
−1 SD	Stress→Reframing→Affect	−0.022	0.020	[−0.068, 0.011]
Optimism				
+1 SD	Stress→Avoidant→Mental Health	**−0.051**	**0.020**	**[−0.094, −0.016]**
Mean	Stress→Avoidant→Mental Health	−0.023	0.018	[−0.060, 0.010]
−1 SD	Stress→Avoidant→Mental Health	−0.007	0.020	[−0.047, 0.035]
Optimism				
+1 SD	Stress→Humor→Mental Health	0.018	0.015	[−0.007, 0.051]
Mean	Stress→Humor→Mental Health	−0.003	0.013	[−0.032, 0.023]
−1 SD	Stress→Humor→Mental Health	−0.015	0.016	[−0.054, 0.013]
Resilience				
+1 SD	Stress→Escapist→Affect	**−0.048**	**0.024**	**[−0.105, −0.010]**
Mean	Stress→Escapist→Affect	−0.027	0.018	[−0.068, 0.001]
−1 SD	Stress→Escapist→Affect	−0.011	0.017	[−0.048, 0.022]
Resilience				
+1 SD	Anxiety→Escapist→Affect	**−0.073**	**0.025**	**[−0.126, −0.030]**
Mean	Anxiety→Escapist→Affect	**−0.059**	**0.020**	**[−0.102, −0.024]**
−1 SD	Anxiety→Escapist→Affect	**−0.048**	**0.018**	**[−0.088, −0.018]**
Resilience				
+1 SD	Stress→Avoidant→Mental Health	**−0.055**	**0.020**	**[−0.099, −0.019]**
Mean	Stress→Avoidant→Mental Health	−0.022	0.016	[−0.055, 0.006]
−1 SD	Stress→Avoidant→Mental Health	0.004	0.018	[−0.031, 0.040]
Resilience				
+1 SD	Stress→Humor→Mental Health	0.020	0.015	[−0.005, 0.055]
Mean	Stress→Humor→Mental Health	−0.002	0.012	[−0.027, 0.023]
−1 SD	Stress→Humor→Mental Health	−0.019	0.015	[−0.055, 0.006]
Resilience				
+1 SD	Anxiety→Humor→Mental Health	**0.031**	**0.014**	**[0.008, 0.061]**
Mean	Anxiety→Humor→Mental Health	**0.021**	**0.010**	**[0.005, 0.043]**
−1 SD	Anxiety→Humor→Mental Health	0.013	0.009	[−0.002, 0.034]
Hope				
+1 SD	Anxiety→Media Exp.→Flourishing	−0.011	0.015	[−0.049, 0.012]
Mean	Anxiety→Media Exp.→Flourishing	0.007	0.012	[−0.014, 0.033]
−1 SD	Anxiety→Media Exp.→Flourishing	0.020	0.016	[−0.005, 0.058]

*Unstandardized coefficients. Indices of moderated mediation are reported in-text. Significant mediation at a given moderator level is indicated by bold and a 95% confidence interval that excludes zero. Optimism and resilience effects are in interaction with the IV (stress or anxiety). Hope effect is in interaction with the mediator (media exposure). Parallel mediation models with controls.’*

Resilience moderated the effect of stress on affect via escapist coping, *index* = −0.025, *SE* = 0.014, 95% CI [−0.058, −0.002]. Resilient people (1 *SD* above the mean optimism score) had a positive effect of stress on escapism which was then linked to negative affect. Resilience also moderated a similar effect of anxiety on affect via escapist coping, *index* = −0.016, *SE* = 0.010, 95% CI [−0.038, −0.001]. Although anxiety was generally associated with more escapism, this relationship was stronger among more resilient individuals. The result of this interest in escapist coping was less positive affect.

Resilience also moderated the indirect effect of stress on mental health via avoidant coping, *index* = −0.039, *SE* = 0.014, 95% CI [−0.069, −0.014]. In other words, stressed yet resilient people showed more avoidant media coping behaviors, which were associated with reduced levels of positive mental health. In contrast, stressed yet resilient individuals also sought more humor, but this was somewhat beneficial for their mental health, *index* = 0.026, *SE* = 0.013, 95% CI [0.006, 0.054]. Resilience also interacted with anxiety to produce an indirect effect on mental health via humor, *index* = 0.012, *SE* = 0.007, 95% CI [0.000, 0.029]. Moderate and high resilience (i.e., mean scores or higher) facilitated positive effects of anxiety on humor coping, which benefited mental health, suppressing anxiety’s overall effect.

There was less evidence that traits interacted with media use to influence psychological well-being outcomes in the back half of the model. Neither trait optimism nor resilience moderated effects of media use on affect, mental health, or flourishing. Trait hope did moderate the influence of media exposure on flourishing, *index* = −0.035, *SE* = 0.024, 95% CI [−0.096, −0.0003]. Anxiety was associated with more media exposure, and the effect of this greater quantity of media use on flourishing was negative for very hopeful individuals and positive for very un-hopeful individuals.

## Discussion

In this study, we examined how stress and anxiety during a global pandemic—involving shutdowns and social distancing—related to different patterns of media use among university students, and how that media use was linked to affect, mental health, and flourishing. A survey of students at two American universities, conducted in the immediate weeks after face-to-face study and work were suspended, revealed that stress and anxiety were related to various patterns of media use and in particular a variety of coping strategies using media. In general, we find that students reporting heightened stress and anxiety reported different media-based coping styles, and these were associated with differential relationships with our measures of well-being. Prior literature on media use as a tool for coping tends to paint media use as a monolithic, and often problematic, coping behavior (e.g., Carver and Connor-Smith, 2010; Müller et al., 2016). However, media psychologists have amply demonstrated media may be sought for a variety of uses and may serve a number of diverse gratifications for users (Rubin, 2002). The evidence presented here suggests that using media for coping is not only common, but that different types of media experiences are sought by stressed versus anxious individuals, and that different coping styles associated with these consumption patterns are associated with diverse outcomes relevant for psychological well-being. A summary of findings is presented in Table 7. Partial support was obtained for H1, H2, H4, H5, H6, H7, and H8, and a number of interactions were found for RQ1, RQ2, and RQ3. The only unsupported prediction (H3) failed to show that the quantity of media exposure had any discernable influence on well-being outcomes.

Generally, results suggest that acute stress and anxiety resulting from the COVID-19 situation were associated with an increased tendency to use media as a coping tool, and some (but not all) media coping strategies were associated with positive affect, positive mental health, and flourishing. These results underscore the relevance of media use for coping during the pandemic, and the potential importance of media use as a psychological resource in times of crisis. Further, findings suggest trait resilience, hope, and optimism interact to influence these effects, and that stress and anxiety were both associated with adaptive *and* non-adaptive forms of media coping. In the remainder of this paper, we detail these relationships and how they can inform our understanding of individual responses to stress and anxiety through media coping.

First, we would note that reports of stress and anxiety were very present in our sample, and they were, as predicted, negatively associated with psychological well-being indicators of positive affect, mental health, and (in the case of stress) flourishing. These results underscore the need to understand how students coped with these negative psychological states given the limited physical and social resources available to them during social distancing. The particularities of stress and anxiety provoked by COVID-19 and the associated stay-at-home orders resulted in clear patterns of media use for coping with negative emotions.

Yet, stress and anxiety were differentially associated with unique patterns of media use, including both the media-based coping strategies employed and the entertainment outcomes experienced. Stress was associated with more hedonic media use and less eudaimonic media use than anxiety. Stress was also associated with avoidant and escapist coping via media (but less than anxiety). These results are in line with escapist theories of media use (e.g., Kubey and Csikszentmihalyi, 1990; Moskalenko and Heine, 2003) suggesting that stressed students were attempting to emotionally escape their current stress levels via hedonically pleasant media choices, unrelated to the COVID-19 crisis. We also found that escapist coping via media was associated with less positive affect, and avoidant coping with lower mental health scores. Overall, these results suggest that when stressed, students turned to the media for escape, and to avoid unpleasant associations with the source of their stress, which may be a maladaptive coping technique for overall psychological well-being outcomes. Yet, we would note that stress was not associated with overall increases in media exposure, suggesting that the style of media coping and the type of media used are more relevant to understanding dysfunctional coping via media than the mere quantity of media exposure.

Students experiencing high anxiety, on the other hand, were more likely to report higher overall media exposure, as well as more eudaimonic media use. This appears to be a more adaptive form of media coping, as eudaimonic media was associated overall with more positive affect. Additionally, anxiety provoked multiple types of coping strategies, showing medium-sized positive associations with all five forms of media coping which emerged in our analysis. Although, like stress, anxiety was associated with escapist and avoidant coping, anxious individuals also used media for problem-focused coping as well as to reframe the current situation, and to provide humor and insight. These latter forms of coping via media are of particular interest as they were positively related to our psychological well-being outcomes.

These different patterns of media use seem to suggest that media exposure is used differently in response to the psychological states of stress and anxiety. While students reporting stress and students reporting anxiety both reported using media to cope in short-term ways, such as escapism, anxious individuals were far more likely to report adaptive forms of media coping, such as problem-focused media use. These differences may be due to the ways in which stress and anxiety differ, particularly in terms of duration of the experience. Whereas stress refers to more ephemeral perceptions of situational threat (Cohen et al., 1983), anxiety as conceptualized by Spitzer et al. (2006) refers to more generalized and long-lasting feelings of worry and nervousness. As a consequence, the use of short-term coping strategies such as avoidance and escapism may be particularly appealing for stressed individuals to address this more fleeting state of perceived threat. Anxious individuals, in contrast, seem to demonstrate a twofold strategy: while they too addressed their negative affective state with short-term, emotion-focused coping strategies such as avoidance, escapism, and humor-based coping, they also use media for problem-focused coping, presumably to address the more persistent nature of anxiety.

The fact that anxious individuals reported problem-focused coping played a role in their media use corresponds with their preference for eudaimonic entertainment. Eudaimonic content, in contrast to hedonic content, frequently provides role models for positive adaptation to critical life events, rather than short-term mood enhancement (e.g., Slater et al., 2018). Perhaps anxious individuals perceive a longer time-frame associated with their stressors, motivating media use which supports both active modes of problem-focused and reframing coping, and inspirational, eudaimonic content. Or perhaps stressed individuals perceive the problems associated with COVID and social distancing are fleeting, leading to an overreliance on short-term mood management techniques. While this interpretation remains speculative, the pattern of results found in the present study suggest that future research on media use and coping will benefit from differentiating between coping attempts in response to stress versus anxiety and acute versus chronic stressors.

The mediation findings emphasize the role of diverse media-based coping strategies in the relationships between stress, anxiety, and psychological well-being. Both reframing and humor coping suppressed the effect of anxiety on negative well-being outcomes, specifically affect and mental health. On the other hand, escapist and avoidant coping styles had negative indirect effects of stress and anxiety on affect and mental health. These findings suggest that differentiating media-based coping styles has the potential to explicate the diverse outcomes associated with media use in times of distress – and potentially address the underlying complexity which drives the conflicting findings associating media use and well-being in other literature. Previous contradictory findings on the role of media use as a coping mechanism may be due to different coping strategies used by the individuals experiencing negative mood states. These findings emphasize the need for future work to further explore the boundary conditions and individual predictors of functional versus detrimental forms of media use for stress coping.

The present study further reveals the important role of trait resiliency factors in individual responses to stress, and the role of media use in the stress-coping context. First, our results replicate the findings of previous research on the beneficial effects of psychological resilience: all three resiliency factors showed negative zero-order correlations with stress, and optimism and resilience also showed negative zero-order correlations with anxiety. Furthermore, all three trait resiliency variables positively predicted all three psychological well-being variables assessed in the present study. In sum, this suggests that individuals high in trait resilience, hope, and optimism were less negatively affected by the COVID-19 related social distancing measures, and more successfully upheld psychological well-being in the face of adversity.

In addition to this general buffer effect, the three trait variables also significantly shaped the way that individuals used media in the coping process. Interestingly, while both optimism and trait resilience were negative predictors of media use for coping, hope showed positive associations with three out of the five media-related coping strategies. The negative associations found between optimism and avoidant coping, and also resilience with escapist and humor coping (Table 5), correspond with previous research demonstrating that media are more frequently used for stress coping when other coping resources are limited (e.g., Reinecke, 2009a, b). This suggests that trait optimism and trait resilience act as internal resources, rendering media use a less relevant or appealing coping tool for these individuals. The positive relationships found between hope and media use for coping may reflect the dynamic interplay of hope and coping. Folkman (2010) proposes that hope and coping with life adversities mutually reinforce each other, and that hope helps the individual to persevere in coping efforts. The positive associations between hope and coping found in the present study may thus suggest that the presence of hope drives and facilitates active coping both generally as well as through media use.

Trait resiliency factors also moderated the relationship between stress, anxiety, and many of the media use variables addressed in the present study. Overall, optimism and trait resiliency intensified the relationship between stress and media use for coping. Optimism also moderated the effect of stress on media exposure. This reveals an interesting pattern: As discussed above, optimism and trait resilience showed negative main effects on media use for coping, presumably because individuals high in these traits experienced less stress and anxiety and thus had a lower need for coping. However, when individuals did experience high levels of stress *despite* scoring highly on trait optimism and trait resilience, they responded more strongly in terms of media-related coping efforts. This may suggest that individuals high in these resiliency traits may generally react more resolutely to perceived stress and that media use is an important tool in these coping efforts. Trait hope, in contrast, did not moderate the relationship between stress and media use. Given the positive main effects of hope on media use for coping, this may suggest that hope generally increases the importance of media use for coping, and not only if a certain threshold level of stress is reached. While these traits seem to play a key role in times of stress, the fact that these traits were less influential in the context of anxiety (only a single moderation effect was found between anxiety and any of the media use variables) underlines the need to clearly differentiate between stress and anxiety in the context of media use for coping.

Finally, the three trait variables also moderated some of the relationships of media use with psychological well-being as well as some of the indirect effect of stress and anxiety on psychological well-being via media use. Resilience factors were generally less likely to moderate effects of media on well-being than they were to moderate effects of stress and anxiety on media use. Pessimists saw helpful effects of their reframing coping on their affective states. Those with high hope experienced less flourishing when anxious, and those with low amounts of hope experienced less flourishing in response to humor.

The moderated mediation effects found for trait optimism and resilience showed mixed patterns, mostly driven by those with lower levels of the resiliency factors. Under high levels of optimism or resilience, stress and anxiety were more likely to lead to avoidant and escapist media use which was harmful for well-being. However, in contrast to that maladaptive coping, those same optimistic or resilient individuals were also more likely to find adaptive coping through humor. Trait variables increased the likelihood for both adaptive and maladaptive media-related coping attempts as a reaction to stress and anxiety, and thus increased both the positive and negative indirect effects of stress on psychological well-being via media coping.

Overall, these results demonstrate that media use and other coping resources, such as the protective and promotive traits addressed in this study, show complex interactions in the context of stress and anxiety, emphasizing the need for future research to explore the boundary conditions of beneficial media effects in response to negative psychological states more systematically. Furthermore, the direction of the relationship between media use and resiliency factors remains an open question. In the present study, resiliency traits were treated as predictors of media use and the resulting relationships with psychological well-being outcomes. However, other research suggests that media use may also have long-term effects on resilience and facilitate or impair the development of psychological traits that facilitate positive adaptation to adversity (Reinecke and Rieger, 2021).

### Limitations

First, we note that the findings presented here are limited by the use of a cross-sectional survey design. Although our theoretically grounded model conceptualizes psychological well-being variables (affect, mental health, and flourishing) as outcomes of media use, it is likely that pre-existing levels of psychological well-being impact media use (cf. Zillmann and Vorderer, 2000) and they may also influence stress and anxiety. Future work should examine longitudinal relationships between these variables to establish causal relationships, when possible. Also, the focus of the study was college students in the United States, however the sample was non-probability, and drawn from two large public universities in different parts of the country, so should not be taken as representative of all American college students. However, mental health problems, and heightened stress in particular, are rampant on American college campuses (Beiter et al., 2015; Francis and Horn, 2017), and prior literature demonstrates media use is a common coping tactic for this audience (Prestin and Nabi, 2020). More broadly, drawing inferences from these data about other populations’ media use and psychological well-being in the wake of the pandemic should be met with caution. However, the COVID-19 crisis and the ensuing policies of social distancing and mass closures impacted people all around the globe. Preliminary reports suggest media demand and pandemic-related media content consumption in particular increased across the United States (Sutton, 2020; Weissbrot, 2020) and elsewhere (Gold, 2020; Szalai and Jarvey, 2020). The results reported here, at a minimum, speak to this broader context, and point to continued avenues for inquiry exploring the variety of ways people use media to cope with new stresses and anxieties.

In regard to our measures, a recent study (Shaw et al., 2020) illustrated that self-reports of media use tend to inflate relationships with psychological well-being variables, compared to unobtrusive tracking of device usage. We attempted to mitigate the limitations of self-reported media use by asking participants about a variety of specific media platforms, and asked them to report average daily hours for each platform in the context of social distancing, however we note this as a limitation. Additionally, our measure did not allow for specific probing into the use of media multitasking, or to separate multitasking from solo media use. We believe that the media exposure scores in our data may in many cases reflect the accumulation of multiple media which were used concurrently. In this way, our measure does validly assess the extent and intensity of media exposure, but less so the precise hours and minutes devoted to media versus non-media activities.

Finally, we would note that some effect sizes in the study were small. We would hesitate to describe small effect sizes as a limitation, as the effect sizes may reflect the true parameter in the population, particularly when dealing with distal effects such as those of trait variables on state appraisals. That said, we would caution overinterpretation of our results where the dataset values are close to zero, without subsequent replication of these findings with a larger sample. Similarly, we would caution that including multiple testing of mediators and moderators in one study may have led to alpha error inflation. Again, future work to replicate these findings is needed, particularly to lend robust estimation to our model parameters. A separate point with regard to effect sizes is the extent to which these effects are practically consequential. Small to medium effect sizes suggest that media played a modest role in university students’ well-being during the initial stage of COVID-19. Media are one piece in the puzzle of coping and well-being, especially during a complex and dynamic situation such as a global pandemic.

## Conclusion

Media may be a productive tool for strategic coping, however it is not a panacea. The findings reported here demonstrate users’ traits and motivations interact with media use behavior to influence functional and dysfunctional outcomes of media-based coping, and results clearly demonstrate a range of coping styles may be associated with media use. Continued exploration of different media-based coping strategies employed by individuals—and their unique contributions to stress and anxiety reduction and increased well-being in times of crisis—may elucidate long-standing conflicting findings relating media use with both detrimental and positive psychological outcomes, and better explicate the ways in which media use may be adaptive or maladaptive, based on users’ individual traits, needs, selections and motivations.

## Data Availability Statement

The dataset presented in this study can be found in an online repository at: https://osf.io/ktwrn.

## Ethics Statement

The studies involving human participants were reviewed and approved by the Human Research Protection Program, Michigan State University, and the Behavioral/NonMedical Institutional Review Board, University of Florida. The participants provided their informed consent to participate in this study.

## Author Contributions

AE contributed ideas, theorizing, data collection, and writing. BJ contributed theorizing, data collection and analysis, and writing. LR contributed to theorizing and writing. SG contributed to data collection and writing. All authors contributed to the article and approved the submitted version.

## Conflict of Interest

The authors declare that the research was conducted in the absence of any commercial or financial relationships that could be construed as a potential conflict of interest.

## Footnotes

1 president.msu.edu

2 coronavirus.ufl.edu

3 Additional open-ended items asked participants to list or describe media content that they had used more, used less, and actively avoided during social distancing, as well as media content they used that was especially hopeful, stressful, connective, depressing, joyful, or guilt-inducing. Analysis of these items will be reported elsewhere.

4 This variable was named “time spent with media” in the pre-registration, but was changed to more accurately reflect the measurement of media exposure.
